# Antibiotic resistance among *Escherichia coli* isolates from stool samples of children aged 3 to 14 years from Ujjain, India

**DOI:** 10.1186/1471-2334-13-477

**Published:** 2013-10-14

**Authors:** Pragya Shakya, Peter Barrett, Vishal Diwan, Yogyata Marothi, Harshada Shah, Neeraj Chhari, Ashok J Tamhankar, Ashish Pathak, Cecilia Stålsby Lundborg

**Affiliations:** 1 Department of Microbiology, R.D. Gardi Medical College, Ujjain, India; 2 Global Health (IHCAR), Department of Public Health Sciences, Karolinska Institutet, Stockholm, Sweden; 3 Department of Public Health and Environment, R.D. Gardi Medical College, Ujjain, India; 4 Department of Community Medicine, R.D. Gardi Medical College, Ujjain, India; 5 Department of Environmental Medicine, R.D. Gardi Medical College, Indian Initiative for Management of Antibiotic Resistance, Ujjain, India; 6 Department of Paediatrics, R.D. Gardi Medical College, Ujjain, India; 7 Department of Women and Children’s Health, International Maternal and Child Health Unit, Uppsala University, Uppsala, Sweden

**Keywords:** *E. coli*, Faecal, Children, Commensal, Antibiotic resistance, Asia

## Abstract

**Background:**

Antibiotic resistance is a major global public health concern, particularly in settings where few treatment options are available. Limited research has been done on antibiotic resistance in *Escherichia coli* of Indian children at community level. Therefore we studied antibiotic resistance patterns in *E. coli* isolates from stool samples of children aged 3-14 years from Ujjain, Central India, to investigate associations of resistance with demographic variables.

**Methods:**

Children, 3-14 years of age, were included from 30 randomly selected villages of Palwa demographic surveillance site, Ujjain, India. Parents were interviewed using a questionnaire, and stool samples were collected from participating children. *E. coli* were isolated from stool samples (n = 529), and susceptibility testing to 18 different antibiotics was done using standard methods.

**Results:**

The proportions of isolates resistant to various antibiotics were, nalidixic acid, (45%), tetracycline (37%), ampicillin (37%), sulfamethoxazole/trimethoprim (29%) and amoxicillin/clavulanic acid (29%). No isolates were resistant to imipenem. Overall, 72% of isolates were resistant to at least one antibiotic and 33% were multi-drug resistant. High rates of cross-resistance were seen for 15 (83%) of the antibiotics studied. *E. coli* isolates from children with literate mothers were more resistant to penicillins and fluoroquinolones. ESBL-producers comprised 9% of the isolates.

**Conclusion:**

Antibiotic resistance and cross-resistance were common in *E. coli* from stools of children. Resistance rates were associated with maternal literacy.

## Background

Antibiotic resistance is a major global public health concern [1], particularly in settings where few treatment options are available, either due to lack of availability or affordability of second line therapies. Commensal *Escherichia coli* can act as reservoirs of resistance genes in the human gut. These resistant genes might be rapidly transferred to other commensal or pathogenic organisms [2,3]. Faecal *E. coli* is regarded as a useful indicator of the spread of acquired antibiotic resistance genes in the community [4,5].

Limited research has been done with regards to antibiotic resistance in *E. coli* among Indian children. In the few studies conducted, wide variation has been demonstrated in resistance rates of *E. coli* isolates from reportedly healthy children [6,7]. In the geographical area of the present study, high rate of broad-spectrum antibiotic prescribing for self-limiting conditions has been shown among children [8,9]. The high rate of antibiotic prescribing is likely to result in high rates of resistance. The main aim of this study was to describe prevalence of resistance in *E. coli* isolates from reportedly healthy children in the community, towards antibiotics commonly used locally. The secondary aim was to investigate associations of resistance with demographic variables.

## **Methods**

**Figure 1:**
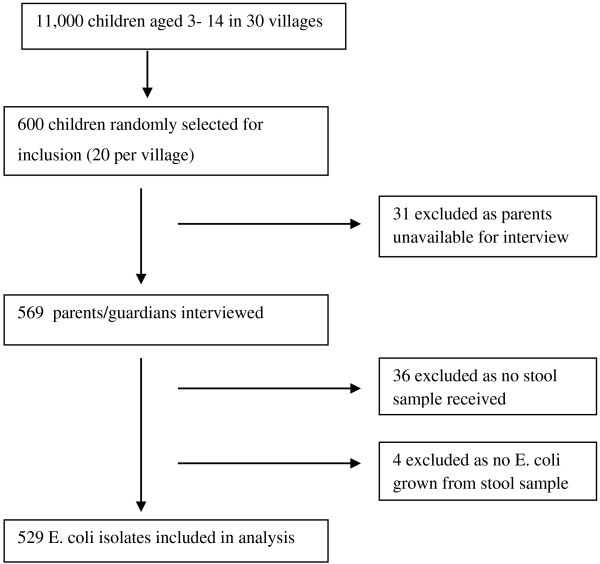
Study design.

This cross-sectional study was conducted in the Palwa demographic surveillance site (DSS) of Ruxmaniben Deepchand Gardi Medical College (RDGMC), Ujjain, Central India, between January and March 2011. The district of Ujjain has a population of almost two million people and 61% live in rural area [10]. The DSS contains a total of 60 villages, and has been described elsewhere [11]. Computer generated random numbers were used to select 30 villages from the DSS. The study design is summarised in Figure 1. Healthcare in Ujjain district is provided in public hospitals, charitable hospitals and private clinics. There are also large numbers of non-allopathic practitioners from traditional medical systems, and informal healthcare providers (IHP). Most qualified healthcare professionals work in the private sector in urban areas, whereas most of the IHP work in rural areas [12,13].

Children aged between 3 – 14 years were included in the study. OpenEpi software was used to calculate a required sample size of 372, given an anticipated frequency of resistance to at least one antibiotic of 50%, with 95% confidence interval [14]. The actual number of participants sought exceeded the sample size due to concerns of non-participation and to try to ensure a minimum of 10 stool samples obtained per village. One child per family was included in the study.

Trained research assistants visited selected children’s homes. They discussed the study with the children’s parents/guardians, invited them to participate and informed about the right to withdraw at any time and assured confidentiality. Parents who consented for their children to participate were included (Figure 1). Parents were interviewed using a structured questionnaire, which included questions on demographic details of the child and family, recent illness among the child and health-seeking behaviour in the event of illness. Research assistants provided sterile stool sample collection containers and collected the samples the following morning from each home.

All stool samples were transported to microbiology laboratory at RDGMC. Processing was started within four hours of sample collection. Stool samples were streaked on MacConkey agar for isolation of *E. coli.* Colonies morphologically resembling *E. coli* were confirmed by using biochemical identification tests [15]. *E. coli* isolate from each stool sample [16] was subjected to antimicrobial susceptibility testing using Kirby-Bauer disc diffusion method. CLSI interpretative criteria for susceptibility and resistance were used [17].

ATCC *E. coli* 25922 was simultaneously tested as control with each batch of antimicrobial susceptibility testing performed. After verifying the results of the standard strain, test sample results were interpreted. Two microbiologists read zone diameters independently by using antibiotic zone scale (Himedia, Mumbai, India).

Antibiotic susceptibility was tested for antibiotics commonly used in healthcare facilities and in the community in Ujjain district. Multi-drug resistance (MDR) was defined as resistance of an isolate to any antibiotic from at least three different antibiotic groups [18]. Extended-spectrum beta-latamases (ESBLs) were phenotypically detected by the combined disc diffusion method with cefotaxime (30 μg) and cefotaxime/clavulanic acid (30/10 μg) and ceftazidime (30 μg) and ceftazidime/clavulanic acid (30/10 μg) [17]. *Klebsiella pneumoniae* ATCC 700603 was used as control for testing ESBL production.

Questionnaire responses and drug susceptibility data were collected, cleaned and entered in to IBM SPSS Statistics 20.0 (SPSS Inc., Chicago, IL, USA). Data were analysed using descriptive statistics, frequencies and bivariate analyses (cross-tabulations). A significance level of *p* = 0.05 was used. Associations were determined between socio-demographic variables and health-seeking behaviour with the outcomes (i) resistance to one antibiotic, and (ii) MDR. Associations were first tested using chi-squared tests. Those variables which approached statistical significance (p < 0.2) were entered in to multivariate logistic regression models with backward elimination. Age was used as a covariate. Independent variables used were: sex (male versus female), family type (joint versus nuclear), number of family members (up to five versus six or more), economic status (above poverty line versus below poverty line), maternal education (no education versus at least one year education), maternal occupation (exclusively homemaker versus any other work), paternal occupation (exclusively agriculture versus any other work), acute illness in child e.g., upper respiratory tract infections, diarrhea etc. (yes versus no), and recent antibiotic prescription or remaining unused antibiotic verified at household (yes versus no).

The study was approved by the Ethics Committee of RDGMC, Ujjain, India (114/2010).

## Results

Table 1 shows the demographic details of the families of the 529 children from whom isolates were obtained. The median age of the included children was 9 years. Thirty percent (n = 159) of them had been reported ill in the three weeks prior to the study. Among the children that were reported ill 26% (n = 42) had received an antibiotic.

**Table 1:** Demographic details of participants and their families

		***N = 529***	
		**n**	**(%)**
Sex of children	Male	304	58
	Female	225	42
Family type^a^	Nuclear	215	41
	Joint	314	59
Economic status	Below poverty line^b^	226	44
	Above poverty line	228	56
Caste^c^	Scheduled caste	167	32
	Scheduled tribe	4	1
	Other backward caste	164	31
	Other	194	36
Number of family members	Up to 5 members	199	38
	6 or more members	330	62
Paternal education	Illiterate	111	21
	1-5 years education	183	35
	≥ 6 years education	235	44
Maternal education	Illiterate	344	65
	1-5 years education	138	26
	≥ 6 years education	47	9
Paternal occupation	Agricultural work	328	62
	Labour work	153	29
	Other work	48	9
Maternal occupation	Housework only	233	44
	Housework + Labour work	147	28
	Housework + Agricultural work	145	27
	Other work	4	1

^a^Nuclear family refers to only parents and children living in one household, i.e. a home sharing one kitchen. Joint family refers to parents and children and any other related or unrelated persons living in one household, i.e. a home sharing one kitchen.

^b^Those classified as living below the poverty line were those living in families in possession of a 'Below poverty line’ ration card issued by the Government of India.

^c^Scheduled castes, backward castes and scheduled tribes are groups in society who were historically subject to social disadvantage and exclusion. They are awarded special status by the Constitution of India and receive certain social benefits. (Government of India. Ministry of Home Affairs. Office of the Registrar General & Census Commissioner 2012).

Among the 529 *E. coli* isolates from 529 children, resistance to at least one antibiotic was observed in 72% of isolates (n = 328). MDR was found in one third of isolates (33%, n = 174). ESBL-producers comprised 9% (n = 48) of isolates.

The rates of resistance observed for individual antibiotics, and selected combinations of antibiotics, are shown in Table 2. Table 3 summarises demographic variables, which were significantly associated with resistance to selected individual antibiotics. Isolates from children of mothers with at least one year of education were more resistant to norfloxacin [OR 2.3, 95% CI 1.3 – 4.3; p < 0.01], ciprofloxacin [OR 2.8, 95% CI 1.4 – 5.6; p < 0.01], piperacillin [OR 1.7, 95% CI 1.1 – 2.5; p = 0.013], ampicillin [OR 1.6, 95% CI 1.1 – 2.4; p = 0.017] and amoxicillin/clavulanic acid [OR 1.8, 95% CI 1.2 – 2.7; p < 0.01]. Paternal education was not associated with resistance in commensal *E. coli* of children*.* Isolates obtained from girls were more resistant to tetracycline compared to isolates from boys [OR 1.5, 95% CI 1.1 – 2.2; p = 0.025].

**Table 2:** **Prevalence of resistance of *E. coli *to individual antibiotics and to combination of selected antibiotics from different groups**

	***N = 529***	
**Antibiotic**	**n**	**(%)**
Tetracycline (A)	197	37
Ampicillin (B)	197	37
Piperacillin	175	33
Amoxicillin/Clavulanic acid	154	29
Piperacillin/Tazobactam	12	2
Ciprofloxacin	40	8
Norfloxacin	57	11
Nalidixic acid (C)	239	45
Cotrimoxazole (D)	154	29
Cefoxitin	22	4
Cefotaxime	70	13
Ceftazidime	72	14
Ceftriaxone (E)	71	13
Cefepime	71	13
Imipenem	0	0
Gentamicin	22	4
Amikacin	26	5
Chloramphenicol	23	4
A + B	110	21
A + B + C	72	14
A + B + C + D	62	12
A + B + C + D + E	25	5

**Table 3:** **Prevalence (%) of resistance of *E. coli *to selected antibiotics associated with demographic variables**

		**TET**	**AMP**	**AMC**	**FEP**	**CIP**	**NOR**	**ADR**	**MDR**
Sex of child	Female	43*(**)	41	31	17 *	9	12	75	36
	Male	**33**	35	28	**11**	7	10	69	31
Family type	Joint	39(**)	35	29	13	7	14	72	33
	Nuclear	**34**	41	29	14	8	9	72	34
Maternal education	>1 year	38	43(**)	35*(**)	16	13*(**)	16*(**)	72	36
	0 years	36	**34**	**26**	12	**5**	**8**	72	31
Given antibiotics (last three weeks)	Yes	50	43	38	19	12(**)	12	81	36
	No	36	37	28	13	**7**	11	71	33

Associations of antibiotic resistance Percentage (%) prevalence of resistance shown for each antibiotic according to selected demographic factors. (TET = tetracycline; AMP = ampicillin; AMC = amoxicillin/clavulanic acid; FEP = cefepime; CIP = ciprofloxacin; NOR = norfloxacin; ADR = resistance to any antibiotic; MDR = multi-drug resistance).

*Statistically significant by chi-squared analysis.

**Statistically significant by multivariate logistic regression analysis, adjusted for age.

Prevalence of resistance to any antibiotic varied widely between different villages, from 40 - 100% of isolates, and prevalence of MDR also varied from 0 - 58%. Children who had been ill within three weeks prior to the study had slightly higher rates of carriage of *E. coli* which were resistant to at least one antibiotic (74% vs. 71%), and also of MDR (38% vs. 31%), but the differences were not statistically significant. Children who had received an antibiotic within the previous three weeks had higher odds of carriage of *E. coli*, which were resistant to ciprofloxacin [OR 9.1, 95% CI 1.6 – 50.6; p = 0.012].

Age was not found to be significantly associated with resistance to individual antibiotics or MDR. Other variables analysed in relation to antibiotic resistance were caste, economic status, number of family members, paternal and maternal occupation, and health-seeking behaviour in the event of child illness. None of these variables were significantly associated with resistance of *E. coli* isolates.

## Discussion

### Resistance patterns in children in Ujjain district

To our knowledge, this is the first community-based study, which describes the prevalence of antibiotic resistance among *E. coli* isolated from reportedly healthy children in Central India. The study shows high rates of resistance to individual antibiotics, with *E. coli* isolates from most children (72%) resistant to at least one antibiotic. This differs from previous resistance rates seen in *E. coli* isolates from children from southern (63%) and eastern India (38-68%) [6,7]. Geographical variation in resistance patterns has been reported previously [7,19].

High rates of antibiotic prescribing have been reported from hospitals in the same geographical area [19]. There are about 475 private pharmacies in Ujjain where clients can buy drugs. Antibiotics are often dispensed without any prescription, and often by those who lack formal qualifications as pharmacists [19]. Antibiotics are also prescribed or dispensed by non-allopathic practitioners and IHPs [8], although they are not authorised to do so.

*E. coli* isolates in the study showed less susceptibility to first line antibiotics, such as penicillins, nalidixic acid, cotrimoxazole and tetracycline, which tend to be more affordable and accessible to families. Cross-resistance was also common in the isolates, and prevalence of MDR exceeded that seen elsewhere in India [6,7].This may be indicative of an evolving resistance gene pool in commensal *E. coli* in India. The resistance patterns are unsettling as they relate to healthy children, whereas generally resistance rates of *E. coli* isolates tend to be higher for hospitalised children, where pathogenic *E. coli* often dominate [20]. A previous study by our research group in the same area among women attending antenatal clinics documented resistance to at least one antibiotic in 94% of commensal *E. coli* isolates. A total of 109 (15%) isolates were ESBL producing and 35 isolates were MDR (35%) [21].

### Factors affecting resistance patterns

There has been uncertainty over the effect of age on antibiotic resistance [6,7,22,23]. There is also ambiguity regarding the role a child’s sex plays in susceptibility to antibiotics. Some studies have found greater proportion of resistance in *E. coli* isolates from male children [7,23,24], whereas others demonstrated higher figures in *E. coli* isolates from females [25,26].

An association between maternal education and increased antibiotic resistance was seen in the present study. This factor affected particular antibiotic groups, namely penicillins and fluoroquinolones. Those children whose mothers had attended school were more likely to carry *E. coli* resistant to these antibiotic groups. Maternal education has previously been regarded as a proxy indicator for socioeconomic status (SES) of a family [27]. It has been seen that children of higher SES are more likely to receive antibiotics [28] and previous consumption increases risk of antibiotic resistance [29]. Educated women tend to have a more autonomous role in society compared to illiterate women [30]. However, SES was not independently associated with resistance of *E. coli* in this study.

Our findings showed variation in resistance rates in *E. coli* of nearby villages, although there are no marked differences between these villages. Similar variation has been reported in Southern India previously [6]. Access to antibiotics may account for some of the inter-village variation. The majority of rural private pharmacies in Ujjain district are located along main roads [13], and those living within easier reach of these are likely to have higher rates of consumption.

Environmental factors may also play a role in the variation of resistance rates between villages. Most villages in the study area have no centralised water supply. Exposure to environmental contaminants may thus, vary with differences occurring locally in village water supply. Sources of environmental contamination with resistant bacteria include human effluent and farm run-off; these have been detected in community drinking water in low-income countries [7,31]. Hospital effluent has also been found to be a source of antibiotics and antibiotic resistance genes in Ujjain district [32,33]. The handling of water storage containers used in different villages may also play a role in occurrence of differing rates of resistance, as contamination of household water with coliform bacteria occurs mostly after collection from water sources [34].

This study adds to existing knowledge about resistance in commensal *E. coli* in an Indian community setting. The large number of antibiotics included in the study provides a comprehensive overview of resistance pattern in commensal *E. coli.* However, information was not obtained regarding diarrhoeagenic and non-diarrhoeagenic strains of *E. coli* so we cannot distinguish resistance prevalence between these strains. Only one isolate was tested for antibiotic susceptibility for each child, therefore some resistant isolates may have been missed and the true resistance rates may have been underestimated.

## Conclusions

Resistant *E. coli* are highly prevalent in intestinal flora of reportedly healthy children in Ujjain district. ESBL detection was however comparatively low. The resistance rates in this study differ from those previously reported in other parts of the country. There is a need to enhance local and national research and surveillance efforts to monitor resistance trends of commensal *E. coli* in community.

## Consent

Written informed consent was obtained from the patient’s guardian/parent/next of kin for the publication of this study.

## Competing interests

The authors declare that they have no competing interests.

## Authors’ contributions

VD and PS initiated the project. AJT, CSL, NC, AP, HS and YM participated in developing the concepts, the design and the planning of the study. VD coordinated the study. VD and NC carried out the fieldwork. PS, YM and HS planned and carried out the microbiological analysis. PB, VD, AP, AJT and CSL were involved in analysis and interpretation of data. PB and VD prepared first draft. AJT, CSL, VD, NC, YM, HS and AP revised the paper critically for substantial intellectual content. All authors have read and approved the final manuscript.

## Pre-publication history

The pre-publication history for this paper can be accessed here:

http://www.biomedcentral.com/1471-2334/13/477/prepub
